# Ways to Support Mental Health and Mental Well-being of Racialized and Immigrant Communities: A Concept Mapping Study

**DOI:** 10.1192/j.eurpsy.2025.807

**Published:** 2025-08-26

**Authors:** F. Ahmad, L. Culley, N. Baldeo, K. A. Haque, A. Suleman

**Affiliations:** 1 York University; 2 Social Services Network, Toronto, Canada

## Abstract

**Introduction:**

Although there is recent growing attention on mental health and mental well-being across the globe, supports in this area of healthcare can be a challenge for immigrant and racialized groups with frequent experiences of hardship.

**Objectives:**

This study aimed to gather perspectives of immigrants and racialized community members on strategies central to support their mental health and well-being, with the aim of addressing research-to-practice gaps.

**Methods:**

The study was co-designed in collaboration with a Community Action Table in Markham, Ontario, a setting with 93% of residents self-identifying as Canadian visible minorities (i.e., non-Caucasian descent). A mixed method Concept Mapping methodology was used to engage residents, service providers, and policymakers (n = 68) through three phases of data collection and interpretation.

**Results:**

Participants first brainstormed ways to support their mental health and well-being, generating 283 statements in three group sessions. A consolidated list of 68 statements was then prepared by removing duplicates and merging similar ideas. This list was shared with participants in three group sessions for the sorting and rating actvities: each participant made groups of statements based on a shared meaning and labelled the groups; and rated each statement on a scale of 1-5 for its importance and feasibility to act in next six-months to support the mental health and well-being of their community. The sorted and rated data was then analyzed statistically through techniques of similarity index and hierarchical cluster analysis to produce visual maps, which were shared with participants in the interpretation session for review and naming of clusters followed by open discussion. This led to a 9-cluster concept map comprising of Family Wellness, Awareness & Education, Cultural Sensitivity, Social Service Access, Community Building, Socioeconomic, Food Security, Healthcare Access, and Housing Stability. The rating data showed the clusters of Family Wellness, Housing Stability, Healthcare Access, and Awareness & Education were ranked high for the dimension of importance. In terms of feasibility to act in next six-months, the clusters of Awareness & Education and Family Wellness remained among the top three while the clusters of Housing Stability and Healthcare Access scored low – which was discussed by participants as requiring a multi-year action plan with short- and long-term goals.

**Conclusions:**

Overall, participants viewed mental health and well-being as being closely tied to their living and working conditions while also focusing on family wellness and intergenerational dynamics. The gained insights emphasize a need for multi-sectoral response to support the mental health and well-being supports of immigrant and racialized communities.

**Disclosure of Interest:**

None Declared

